# CaliPopGen: A genetic and life history database for the fauna and flora of California

**DOI:** 10.1038/s41597-022-01479-z

**Published:** 2022-07-05

**Authors:** Joscha Beninde, Erin M. Toffelmier, Aarron Andreas, Celina Nishioka, Meryl Slay, Ashley Soto, Justin P Bueno, Germar Gonzalez, Hannah V. Pham, Molly Posta, Jordan L. Pace, H. Bradley Shaffer

**Affiliations:** 1 UCLA La Kretz Center for California Conservation Science, Institute of the Environment and Sustainability, University of California, Los Angeles, CA 90095 USA; 2 Department of Ecology and Evolutionary Biology, University of California, Los Angeles, CA 90095 USA

**Keywords:** Biodiversity, Evolutionary genetics, Molecular ecology, Conservation biology

## Abstract

CaliPopGen is a database of population genetic data for native and naturalized eukaryotic species in California, USA. It summarizes the published literature (1985–2020) for 5,453 unique populations with genetic data from more than 187,394 individuals and 448 species (513 species plus subspecies) across molecular markers including allozymes, RFLPs, mtDNA, microsatellites, nDNA, and SNPs. Terrestrial habitats accounted for the majority (46.4%) of the genetic data. Taxonomic groups with the greatest representation were Magnoliophyta (20.31%), Insecta (13.4%), and Actinopterygii (12.85%). CaliPopGen also reports life-history data for most included species to enable analyses of the drivers of genetic diversity across the state. The large number of populations and wide taxonomic breadth will facilitate explorations of ecological patterns and processes across the varied geography of California. CaliPopGen covers all terrestrial and marine ecoregions of California and has a greater density of species and georeferenced populations than any previously published population genetic database. It is thus uniquely suited to inform conservation management at the regional and state levels across taxonomic groups.

## Abstract (summary)

**Table:** 

Measurement(s)	genetic variation
Technology Type(s)	DNA sequencing
Factor Type(s)	Kingdom • Phylum • TaxonGroup • MarkerType • SampleSize • GeneTarget • NumMarkers • YearStart • YearEnd • PopName • LongitudeDD • LatitudeDD • CoordError • HabitatType • Lifespan • Fecundity • LifetimeReprodOutput • AgeSexMat • NumBreedingEvents • ReprodMode • BodyLength • AdultMass • CANativeStatus • CESAStatus • SSCStatus • ESAStatus • LifeCycle • AdultHeight • SelfCompatibility • MonoeciousDioecious • Asexual • PollinationMode • SeedDispMode • MassPerSeed • CAEndemicStatus
Sample Characteristic - Organism	eukaryota
Sample Characteristic - Location	California

## Background & Summary

The CaliPopGen database consists of four datasets that contain estimates of population genetic diversity, differentiation, and life history traits for 448 eukaryotic species sampled across California, USA. The state has exceptionally high plant and animal biodiversity, and a correspondingly large number of endangered taxa^1^. It is often divided into 19 terrestrial and three marine ecoregions, reflecting its tremendous geologic and ecological diversity^2,3^, including the highest and lowest elevations in the contiguous U.S., extreme deserts and temperate rainforests, and mean annual precipitation ranging from 150 mm–1200 mm^4^. California is the most populous state in the USA, accommodating roughly 12% of the nation’s human population; the third largest state geographically, encompassing 5% of the country’s continental land area; and is a major agricultural producer. This combination of high species richness and human-mediated pressures constitute a persistent threat to the short- and long-term persistence of biodiversity, and has led to California’s inclusion as one of only two global biodiversity hotspots in the USA^5,6^. Perhaps unsurprisingly, California has the greatest number of documented and possibly extinct species of vascular plants^7^, and more than twice as many federally protected species (total of 287) as any other state^8^. It has also been the focus of more population genetic studies, including states with similarly high numbers of threatened/endangered species like Florida and Hawaii^1^. However, this wealth of genetic information has never been adequately summarized or made publicly available. The few broadly comparative analyses for California have largely been based on inferences derived from fewer than 10 species^9–12^, with the exceptions of one review^13^, and one empirical study^14^, both of which were restricted to marine taxa. California is a perennial leader in biodiversity management, and our compilation of genetic data for the state aligns with the administrative level at which environmental legislation and biodiversity management is implemented, increasing the likelihood that the CaliPopGen database will inform conservation actions.

**Fig. 1:**
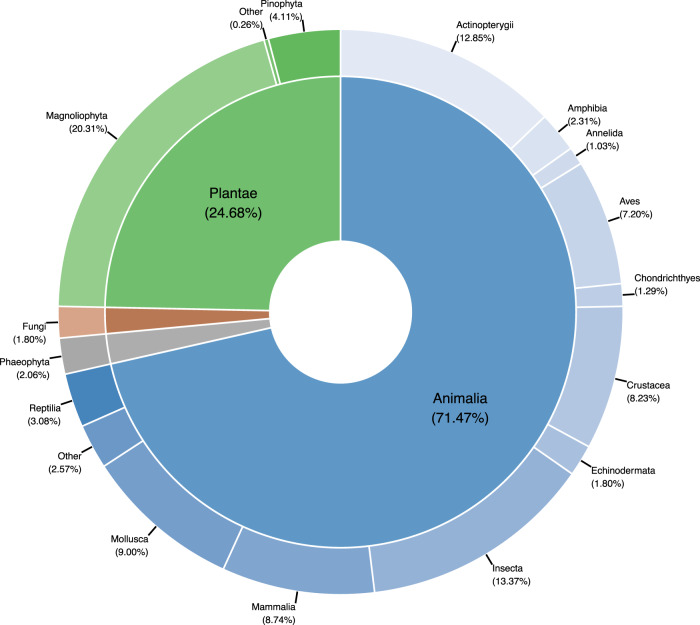
Taxonomic breakdown of species represented in the CaliPopGen database. Values in parentheses represent the total number of species as a percentage of the number of unique species in the database.

**Fig. 2:**
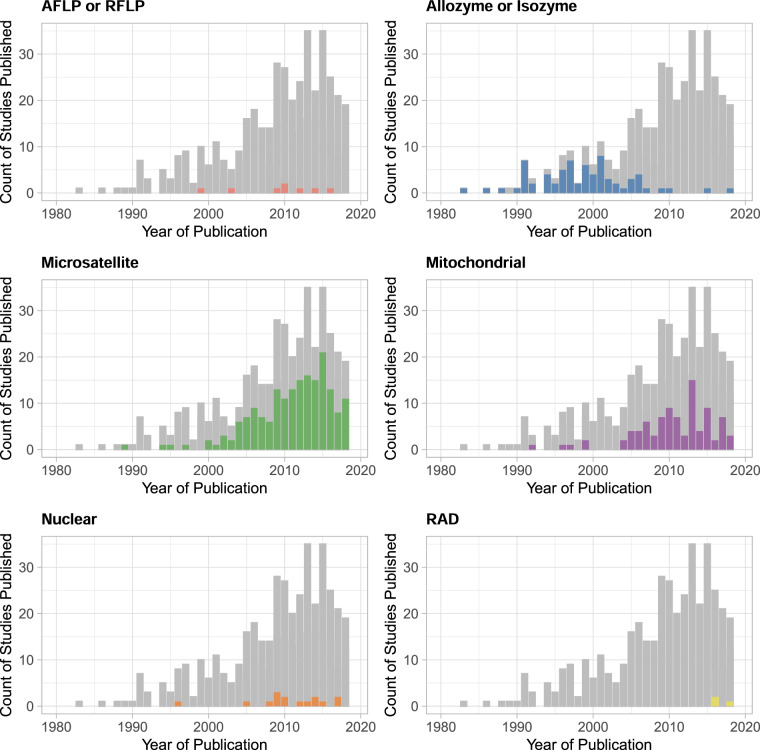
The six predominant marker types included in the CaliPopGen database, demonstrating different publication trends through time. The grey bars in each panel are the total number of published studies across all marker types (and are the same in each panel).

In compiling CaliPopGen, we examined 4,942 published studies identified by our search criteria in the Web of Science, of which 450 met our final inclusion criteria and are included in the database. The majority of genetic samples represented in this database were collected from 1995–2015 (ranging from 1888–2019), and all studies were published between 1983–2020. CaliPopGen contains information on more than 187,394 individuals from 5,453 unique populations, of which 5,276 are spatially georeferenced. These populations include terrestrial (46.6%), marine (21.9%), freshwater (14.1%), amphibious (9.7%), and diadromous (7.7%) populations of fungi (<2% of unique species), chromists (<2%), plants (23%), and animals (73%; Fig. 1). CaliPopGen includes population level data with broader taxonomic coverage than recent, more global compilations, which have focused on freshwater and marine fishes^15^, mammals^16^, mammals and amphibians^17^, vertebrates^18^, and birds, fishes, insects and mammals^19^. Its focus at the regional (state) level is unique. The CaliPopGen database also includes a wider range of molecular markers (Fig. 2), populations and species than these previous population genetic compilations. Molecular markers in our database include RFLPs, AFLPs, allozymes and isozymes, microsatellites, mitochondrial, and other nuclear markers, whereas previously published datasets frequently focussed on one or a few loci (e.g.^15–17,19^) or single marker types (e.g.^18^,). Our inclusion of all available marker types both reflects the change in methodological approaches through time (for example, the temporal replacement of allozymes with microsatellites in the early 2000’s), and presents opportunities for quantitative comparisons among different marker types.

**Fig. 3:**
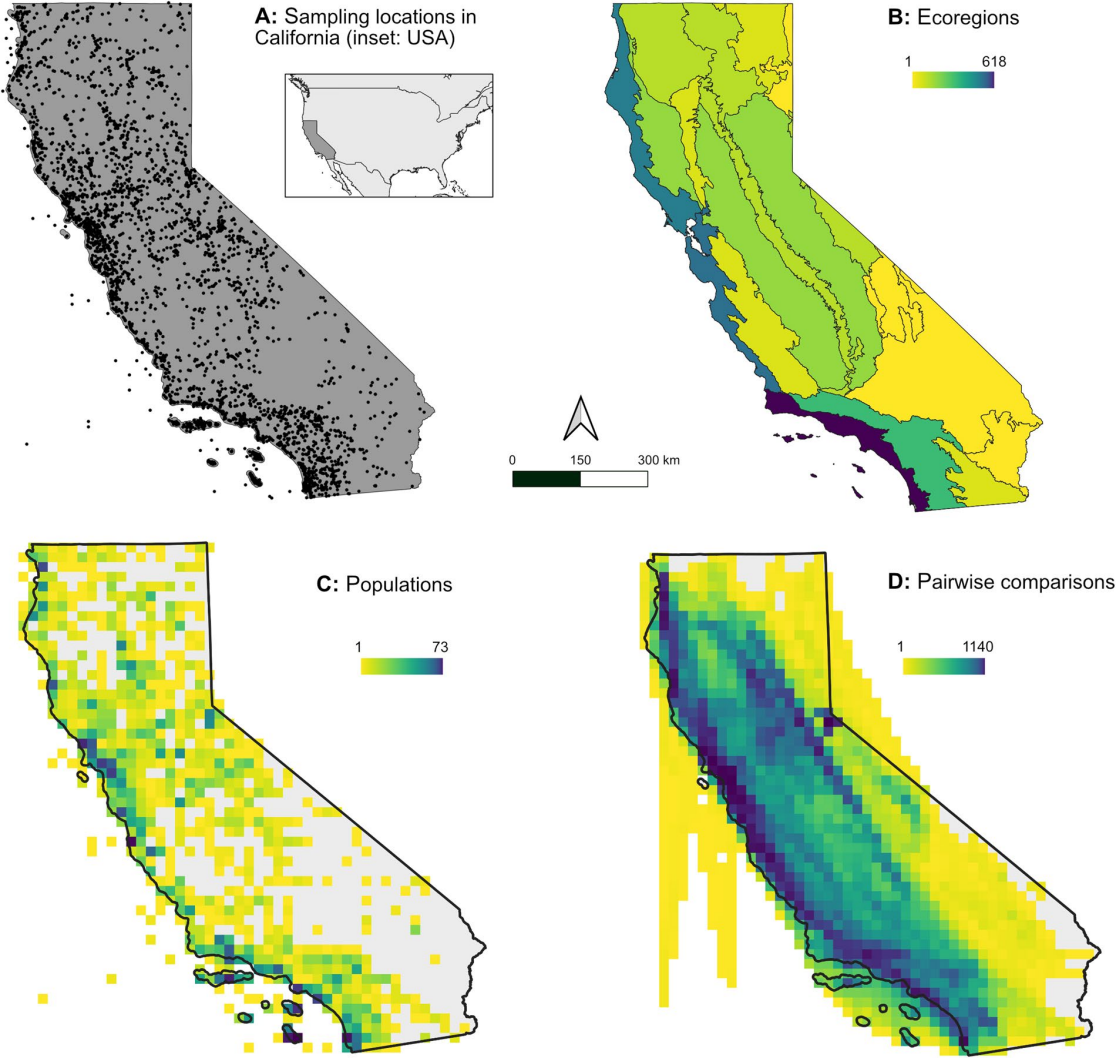
Maps of data contained in the CaliPopGen databases. (**A**) All unique sampling locations of both the population genetic (**Dataset 1**^21^**)** and pairwise comparison (**Dataset 2**^21^**) data**. The inset shows the location of California within the contiguous USA. (**B**) The number of unique populations in CaliPopGen per California ecoregion. Note the relative under-representation of inland desert regions (yellow) and over-representation of coastal ecoregions (purple-blue). (**C**) The number of unique populations of the populations genetic **Dataset 1**^21^ per 20km raster cell. (**D**) The number of straight-line pairwise comparisons of **Dataset 2**^21^ per 20km raster cell.

Expressed as a proportion of the study area, CaliPopGen contains at least an order of magnitude more species (0.83/1,000 km²), populations (9.59/1,000 km²), and individuals (284.04/1,000 km^2^) than the aforementioned studies and databases. This high spatial density of samples across the full ecological scope of California (Fig. 3) should facilitate future analyses of ecological trends at the population level where biological processes actually occur, and is well suited to help identify important mechanisms shaping genetic diversity, connectivity and fragmentation. CaliPopGen should also serve as a point of departure for future studies, providing a genetic baseline against which researchers can contrast and quantify future population genetic impacts resulting from changes in climate or land use. As such, CaliPopGen is an historical antecedent to ongoing genomic initiatives to study the diversity and distribution of California’s flora and fauna, including the California Conservation Genomics Project^20^, and other projects using landscape genomic approaches.

To supplement the genetic data in CaliPopGen, we also compiled datasets containing life history information for all plant and animal species in the database, including adult body size, lifespan, reproductive and dispersal traits, and conservation status.

## Methods

### Population genetic data collection from primary data sources

**Fig. 4:**
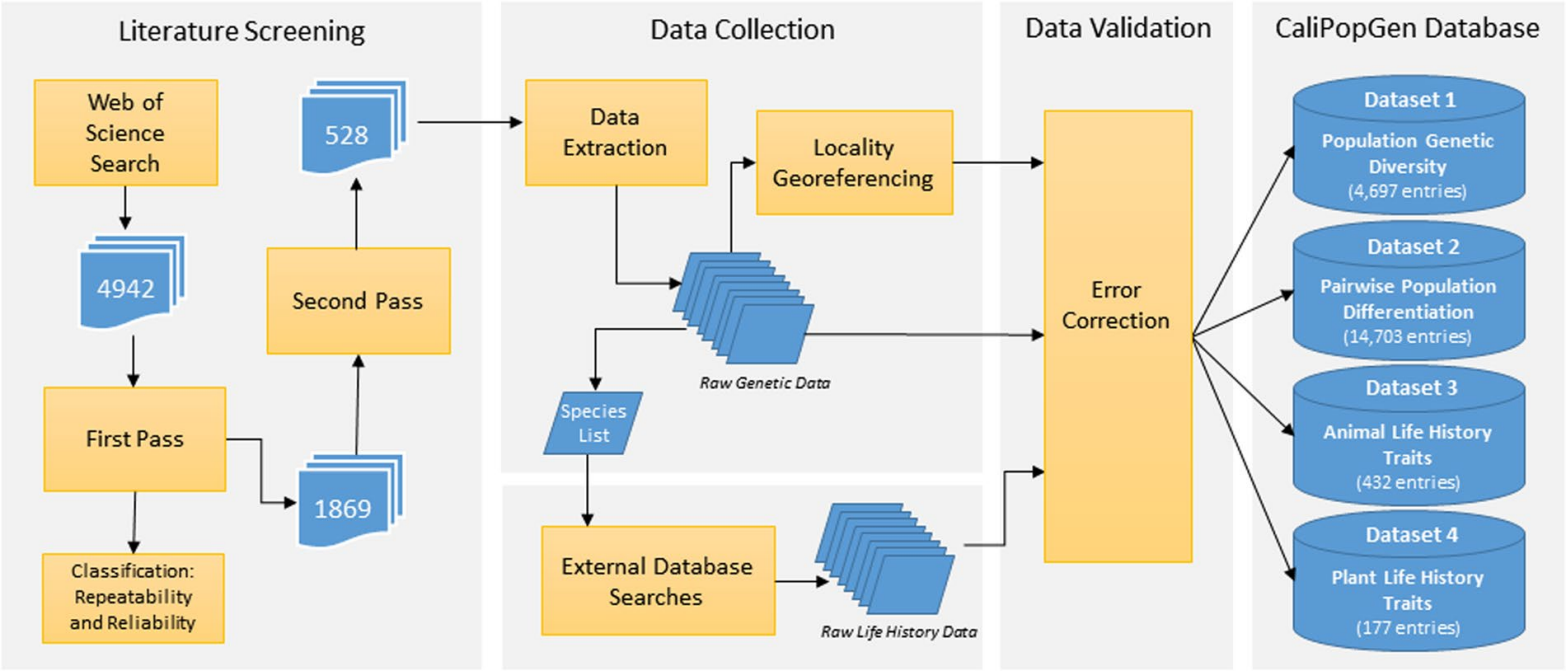
Flow chart of the data collection process that generated the CaliPopGen databases.

Figure 4 describes the overall data collection workflow for the four datasets that comprise CaliPopGen. We first identified literature potentially containing population genetic data for California by querying the Web of Science Core Collection (https://webofknowledge.com/) for relevant literature from 1900 to 2020 with the terms: topic = (California*) AND topic = (genetic* OR genomic*) AND topic = (species OR taxa* OR population*). We included only empirical peer-reviewed literature and excluded unreviewed preprints. In using these search terms, our goal was to broadly identify genetic papers focused on California with population or species-level analyses, while avoiding purely phylogenetic studies or those focused on agricultural or model species. This resulted in 4,942 unique records.

We next screened titles and abstracts to retain articles that: (1) provided data on populations of species which are self-sustaining without anthropogenic involvement; (2) included at least some eukaryote species; (3) included population(s) sampled within California; (4) mentioned measures of genetic diversity or differentiation; and (5) were not reviews (thus restricting our search to only primary literature). We retained 1869 studies after this first pass of literature screening (see Technical Validation for estimate of inter- and intra-screener bias).

Our second, more in-depth screening pass involved reading the full text of these 1869 studies. We had two goals. First, we confirmed that retained papers fully met all five of our inclusion criteria (the first screen was very liberal with respect to these criteria, and many papers failed to meet at least one criterion after close reading). Second, we eliminated papers where the data were not presented in a way that allowed us to extract population-level information. For example, many of the more systematics-focused studies pooled samples from large, somewhat ill-defined regions (“Sierra Nevada” or “Southern California”); if such regions were larger than 50 km in a linear dimension, we deemed them unusable for making geographically-informative inferences. Other studies presented summaries of population data, often in the form of phylogenetic networks or trees, but did not include information on actual population genetic parameters and therefore were not relevant to our database. We retained 528 publications after this second pass.

From this set of papers, we extracted species, locality, and genetic data for each California population or sampling locality described in each study (Fig. 3A**)**. This included Latin binomial/trinomial, English common name, population identifiers, and geographic coordinates of sampling sites. We also noted population/sampling localities that were interpreted as comprised of interspecific hybrids, and listed both parental species. We collected population genetic diversity and differentiation statistics for each unique genetic marker for each population/sampling locality; as a result, a sampling locality may have multiple entry rows, one for each locus or marker type. Parameters extracted for each population/marker combination include sample size, genetic marker type, gene targets, number of loci, years of sampling, and reported values for effective population size (*N*_*e*_), expected (*H*_*E*_) and observed (*H*_*O*_,) heterozygosity, nucleotide diversity (*π*, pi), alleles-per-locus (*APL*), allelic richness (*A*_*R*_), percent polymorphic loci (*PPL*), haplotype diversity (*HDIV*), inbreeding coefficient (e.g. *F*_*IS*_, *F*_*IT*_, *G*_*IS*_), and pairwise population genetic comparison parameters (*F*_*ST*_, *G*_*ST*_, *D*_*ST*_, *Nei’s D*, *Jost’s D*, or *phi*). We note that while there are technical differences between allelic richness and alleles-per-locus, source literature often used the terms interchangeably, and we include the parameters and their values as named in the source. We define marker type as the general category of genetic marker used (e.g., “microsatellite” or “nuclear”), while gene targets are the specific locus/loci (e.g., “COI”). We present these data in two separate datasets, one containing all population-level genetic summary statistics (**Dataset 1**^21^, see Fig. 3C and detailed description in Table 1) and a second for estimates of pairwise genetic differentiation (**Dataset 2**^21^, see Fig. 3D and detailed description in Table 2).

**Table 1:** Description of the population genetic data in **Dataset 1**^21^.

Column ID	Description
CitationID	Unique ID assigned to each source article
EntryID	Unique ID assigned to each unique entry in the entire CaliPopGen database
CitationFull	Citation information
Kingdom	Kingdom classification for the species
Phylum	Phylum classification for the species
TaxonGroup	Broadly categorized taxonomic group
ScientificName	Currently accepted Latin binomial (GBIF)
SubspeciesName	Currently accepted subspecies epithet (GBIF)
CommonName	Currently reported English common name (GBIF)
MarkerType	General category of genetic marker
GeneTarget	Specific genes or markers used
NumMarkers	Number of markers used in the study
SampleSize	Number of samples used to calculate genetic parameters. Value may be a non-integer if a mean number of samples across a set of loci was reported.
YearStart	First year of sample collection
YearEnd	Last year of sample collection
PopName	Population or locality name
LatitudeDD	Latitude in decimal degrees
LongitudeDD	Longitude in decimal degrees
CoordError	Estimated radius of error in kilometers for coordinates georeferenced by us
AllelicRichness	Allelic richness
HetExp	Expected heterozygosity
HetObs	Observed heterozygosity
NucDiversity	Nucleotide diversity, pi
EffectivePopSize	Effective population size
AllelesPerLocus	Alleles per locus
PercentPolyLoci	Percent polymorphic loci
HaploDiv	Haplotype diversity
InbreedingCoefType	Type of inbreeding coefficient reported
InbreedingCoefValue	Value of inbreeding coefficient
SpeciesID	Unique ID assigned to this entry
HabitatType	Marine, Freshwater, Diadromous, Terrestrial, Amphibious. See text for descriptions
Columns 32–70	Animal life history data (see Table 3)
Columns 71–101	Plant life history data (see Table 4)

**Table 2:** Description of the pairwise genetic distance data in **Dataset 2**^21^.

Column Name	Description
CitationID	Unique ID assigned to each source article.
EntryID	Unique ID assigned to each unique entry in the entire CaliPopGen database
CitationFull	Reference information
Kingdom	Kingdom classification for the species
Phylum	Phylum classification for the species
TaxonGroup	Broadly categorized taxonomic group
Pop1ScientificName	Currently accepted Latin binomial (GBIF)
Pop1SubspeciesName	Currently accepted subspecies epithet (GBIF)
Pop1CommonName	Currently reported English common name (GBIF)
Pop1Name	Population or locality name of first site in pairwise comparison
Pop1LatitudeDD	Latitude in decimal degrees of first site
Pop1LongitudeDD	Longitude in decimal degrees of first site
Pop2ScientificName	Currently accepted Latin binomial (GBIF)
Pop2SubspeciesName	Currently accepted subspecies epithet (GBIF)
Pop2CommonName	Currently reported English common name (GBIF)
Pop2Name	Population or locality name of second site in pairwise comparison
Pop2LatitudeDD	Latitude in decimal degrees second site
Pop2LongitudeDD	Longitude in decimal degrees second site
CoordError	Estimated radius of error in kilometers for coordinates georeferenced by us
GenDist	Genetic distance score (*F*_*ST*_*, G*_*ST*_*, D*_*ST*_*, Nei’s D, Jost’s D, phi*)
GenDistMetric	Type of pairwise genetic parameter reported (*F*_*ST*_*, G*_*ST*_*, D*_*ST*_*, Nei’s D, Jost’s D, phi*)
GenDistMetricMethod	Name/citation of specific method used to calculate GenDistMetric (if provided)
MarkerType	General category of genetic marker
GeneTarget	Specific genes or markers used
NumMarkers	Number of markers used
SepAnalyses	When multiple analyses were conducted, the level by which data were split is noted here (e.g. species or sampling year)
SpecialComparionsType	Identifies pairwise comparisons across timescales (“temporal”), at different temporal intervals (“spatio-temporal replicate”), of samples collected before 1920 (“historic”), between species (“interspecific”) or hybrid populations (“hybrid”)
Pop1ComparisonCharacteristic	Characteristic of special comparison
Pop2ComparisonCharacteristic	Characteristic of special comparison
Pop1YearStart	First year of sample collection
Pop1YearEnd	Last year of sample collection
Pop2YearStart	First year of sample collection
Pop2YearEnd	Last year of sample collection
SpeciesID	Unique ID assigned to this entry
HabitatType	Marine, Freshwater, Diadromous, Terrestrial, Amphibious. See text for descriptions
Columns 36–74	Animal life history data (see Table 3)
Columns 75–101	Plant life history data (see Table 4)

All genetic data were extracted directly from the source literature. However, we also updated or added to the metadata for these population genetic values in several ways. We included kingdom, phylum, and a lower-level taxonomic grouping for each species (usually class), and updated scientific and common names based on the currently accepted taxonomy of the Global Biodiversity Information Facility^22^. When geographic coordinates were not provided for a sampling locality, as was frequently the case in the older literature, we used Google Maps (https://www.google.com/maps) to georeference localities based on either in-text descriptions or embedded figure maps guided by permanent landmarks like a bend in a river or administrative boundaries. Because this can only yield approximate coordinates, we recorded estimated accuracy as the radius of our best estimate of possible error in kilometers. If coordinates were provided in degree/minute/seconds, we used Google Maps to translate them to decimal degrees. In cases where coordinates were not provided and locality descriptions were too vague to determine coordinates with less than 50 km estimated coordinate error, we did not attempt to extract coordinates but still provide the genetic data. All coordinates are provided in the web Mercator projection (EPSG:3857). We excluded studies that reported genetic parameter values only for samples aggregated regionally (“Southern California” or “Sierra Nevada”). If marker type was not explicitly included, we classified marker type based on the gene targets reported, if provided.

### Life history trait data collection

To increase the utility of CaliPopGen, we also assembled data on life history traits for all animal (**Dataset 3**^21^) and plant (**Dataset 4**^21^) species contained in **Datasets 1**^21^ **and 2**^21^. We assembled trait data that have previously been shown to correlate with genetic diversity, including those related to reproduction, life cycle, and body size, as well as conservation status (e.g.^23–26^,). Life history data were compiled by first referencing large online repositories, often specific to taxonomic groups, like the TRY plant trait database^27^, and the Royal Botanic Gardens Kew Seed Information Database^28^. If trait data for species of interest were unavailable from these compilations, we conducted keyword literature searches for each combination of species and life history trait, and extracted data from the primary literature. When data were not available for the subspecies or species for which we had genetic data, we report values for the next closest taxonomic level, up to and including family, as available in the literature.

For both animals and plants, we defined habitat types as marine, freshwater, diadromous, amphibious, or terrestrial. Marine species include those that are found in brackish or wetland-marine habitats, as well as bird species that primarily reside in marine habitats. Freshwater species include those that are found in wetland-freshwater habitats, as well as species that primarily reside in freshwater. The diadromous category includes fish species that are catadromous or anadromous. We considered species to be amphibious if they have an obligatory aquatic stage in their life cycle, but also spend a significant portion of their life cycle on land. Terrestrial species were defined as those that spend most of their life cycle on land and are not aquatic for any portion of their life cycle. In a few cases (e.g., waterbirds that are both freshwater and marine, semi-aquatic reptiles), a species could reasonably be placed in more than one category, and we did our best to identify the primary life history category for such taxa. If the taxonomic identity of an entry was hybrid between species or subspecies, this was noted in the speciesID column and no life history data were reported.

The CaliPopGen Animal Life History Traits **Dataset 3**^21^ (description of dataset in Table 3) includes habitat type, lifespan, fecundity, lifetime reproductive success, age at sexual maturity, number of breeding events per year, mode of reproduction, adult length and mass, California native status, listing status under the US Endangered Species Act (ESA), listing status under the California Endangered Species Act (CESA), and status as a California Species of Special Concern (SSC). For some traits, value ranges were recorded–for example, minimum to maximum lifespan. In other cases, we recorded single values and, when available, a definition of this single value, (for example, minimum, average, or maximum lifespan). We report either the range of the age of sexual maturity (minimum to maximum), or a single value, depending on the available literature. For sexually dimorphic species, we report female adult length and weight when available, because female body size often correlates with fecundity. Across animal taxonomic groups, different measures of body size and length measurements are often used, reflecting community consensus on how to measure size. Given this variation, we report the type of length measurement, if available, as Standard Length (SL), Fork Length (FL), Total Length (TL), Snout-to-Vent Length (SVL), Straight-Line Carapace (SLC), or Wingspan (WS).

**Table 3:** Description of the animal life-history data in **Dataset 3**^21^.

Column Name	Description	Total entries
SpeciesID	Unique ID assigned to this entry	432
TaxonGroup	Broadly categorized taxonomic group	432
ScientificName	Currently accepted Latin species binomial (GBIF)	432
SubspeciesName	Currently accepted subspecies epithet (GBIF)	88
CommonName	Currently reported English common name (GBIF)	372
HabitatType	Marine, Freshwater, Diadromous, Terrestrial, Amphibious. See text for descriptions	429
LifespanMin	Minimum value for reported lifespan range	90
LifespanMax	Maximum value for reported lifespan range	131
LifespanOther	Value of lifespan if not reported as a range	147
LifespanOtherType	Value type of “LifespanOther” (average, minimum or maximum)	147
Fecundity	The number of offspring or eggs per reproductive event	216
LifetimeReprodOutput	Total lifetime reproductive output	24
AgeSexMatMin	The minimum age for an individual to reach sexual maturity, in years	92
AgeSexMatMax	The maximum age for an individual to reach sexual maturity, in years	79
AgeSexMatOther	Single values for age of sexual maturity in years if not reported as a range	121
AgeSexMatOtherType	Value type of “AgeSexMatOther” (average, minimum or maximum)	121
NumBreedingEvents	Number of breeding events per year	146
ReprodMode	Mode of reproduction (asexual, sexual, both)	312
BodyLength	Adult body length reported in centimeters (cm)	333
BodyLengthType	Adult body length measurement type: SL (standard length) or PCL (precaudal standard length), FL (fork length), TL (total length), WS (wingspan), SCL (straight-line carapace), SVL (snout-to-vent length)	254
BodyLengthSex	The gender of the adult length reported	248
AdultMass	Adult mass, reported in kilograms (kg)	178
AdultMassSex	The gender of the adult mass reported	124
CANativeStatus	Native/non-native: whether the species is known to be native to California	329
CESAStatus	California Endangered Species Act listing status, if any	39
SSCStatus	California Species of Special Concern listing status, if any	49
ESAStatus	Federal Endangered Species Act (ESA) listing status, if any	52
TaxonDataLevel	The taxonomic level at which collected data was obtained, if not for the species or subspecies in question	16
SpeciesSynonyms	List of species synonyms used to acquire information (GBIF)	15
Columns 30–45	Reference sources for trait data	

The CaliPopGen Plant Life History Traits **Dataset 4**^21^ (description of dataset in Table 4) includes habitat type, lifespan, life cycle, adult height, self-compatibility, monoecious or dioecious, mode of reproduction, pollination and seed dispersal modes, mass per seed, California native status, NatureServe^29^ element ranks (global and state ranks, see Table 5 for definitions), listing status under the Federal Endangered Species Act (ESA), and listing status under the California Endangered Species Act (CESA). In contrast to most animal species, plant lifespan was typically reported as a single value. We define life cycles as the following: Annual: completes full life cycle in one year; Biennial: completes full life cycle in two years; Perennial: completes full life cycle in more than two years; Perennial-Evergreen: perennial and retains functional leaves throughout the year; Perennial-Deciduous: perennial and loses all leaves synchronously for part of the year. Some species are variable (for example, have annual and biennial individuals), and in those cases we attempted to characterize the most common modality.

**Table 4:** Description of the plant life-history data in **Dataset 4**^21^.

Column Name	Description	Total entries
SpeciesID	Unique ID assigned to this entry	177
TaxonGroup	Broadly categorized taxonomic group	177
ScientificName	Currently accepted Latin binomial (GBIF)	177
SubspeciesName	Currently accepted subspecies epithet (GBIF)	34
CommonName	Currently reported English common name (GBIF)	144
HabitatType	Marine, Freshwater, Terrestrial. See text for descriptions	116
Lifespan	Reported only for perennial species. Maximum lifespan value reported or highest value of reported lifespan range (years)	61
LifeCycle	Annual, Biennial, Perennial, Perennial-Evergreen, Perennial-Deciduous. See text for descriptions	152
AdultHeight	Maximum height value reported or highest value of reported height range in meters (m)	145
SelfCompatibility	Indicates whether species is self-compatible	98
MonoeciousDioecious	Monoecious: individuals bear both male and female flowers; Dioecious: individuals bear either male or female flowers, but not both	78
Asexual	Indicates whether primary mode of reproduction is asexually	21
PollinationMode	Primary pollination mode: wind, animal, water	120
SeedDispMode	Seed dispersal mode: wind, animal, gravity, water, human	94
MassPerSeed	Fecundity as measured by mass per seed in milligrams (mg)	83
CANativeStatus	Native/non-native: whether the species is known to be native to the state of California	164
CAEndemicStatus	Endemic, near-endemic or distributed only in California & Baja California	62
InvasiveRating	California Invasive Plant Council rating of invasiveness (non-native species only)	27
CESAStatus	California Endangered Species Act listing status, if any	18
CNDDBStatus	Heritage rank as defined by the California Natural Diversity Database. See Table 5 for ranking descriptions.	139
ESAStatus	Federal Endangered Species Act (ESA) listing status, if any	19
TaxonDataLevel	The taxonomic level at which collected data was obtained, if not for the species or subspecies in question	63
SpeciesSynonyms	List of species synonyms used to acquire information (GBIF)	21
Columns 24–37	Reference sources for trait data

**Table 5:** Description of the Conservation status (Heritage Rank) from California Natural Diversity Database^29^.

Global/State rank	Description
GX/SX	Presumed extirpated
GH/SH	Possibly extirpated; known only from historical occurrences but there is still some hope of rediscovery.
G1/S1	Critically imperiled; at very high risk of extirpation in the jurisdiction due to very restricted range, very few populations or occurrences, very steep declines, severe threats, or other factors.
G2/S2	Imperiled; at high risk of extirpation in the jurisdiction due to restricted range, few populations or occurrences, steep declines, severe threats, or other factors.
G3/S3	Vulnerable; at moderate risk of extirpation in the jurisdiction due to a fairly restricted range, relatively few populations or occurrences, recent and widespread declines, threats, or other factors.
G4/S4	Apparently secure; at a fairly low risk of extirpation in the jurisdiction due to an extensive range and/or many populations or occurrences, but with possible vii cause for some concern as a result of local recent declines, threats, or other factors.
G5/S5	Secure; at very low or no risk of extirpation in the jurisdiction due to a very extensive range, abundant populations or occurrences, with little to no concern from declines or threats.

The Global rank (G rank) is a reflection of the overall status of a species throughout its global range. The State rank (S rank) is assigned much the same way as the Global rank, but State ranks refer to the imperilment status only within California’s state boundaries.

Because of the paucity of data available for chromists and fungi, we did not extract life history trait data for the relatively few species in these taxonomic groups.

### Data visualization and summary

We used the R-package raster (v3.1–5) to visualize the spatial extent of the data in CaliPopGen in Fig. 3. Panel (A) shows a summary plot of all unique populations of both the Population Genetic Diversity in **Dataset 1**^21^ and the Pairwise Population Differentiation in **Dataset 2**^21^. Panel (B) shows the total number of unique populations in each California terrestrial ecoregion. Panel (C) depicts all data entries of Population Genetic Diversity **Dataset 1**^21^, summed for each 20x20 km grid cell. Panel (D) shows the density of pairwise straight lines drawn between pairs of localities in the Pairwise Population Differentiation **Dataset 2**^21^, depicted as the total number of lines per 20x20 km grid cell. The number of populations and species of both **Datasets 1**^21^ **& 2**^21^ are summarized for each marine and terrestrial ecoregion in Table 6.

**Table 6:** Summary of total numbers of populations and species per California ecoregion, separately for population genetic and pairwise datasets.

Ecoregion	type	area (km²)	N species PopGen	N populations PopGen	N species Pairwise	N populations Pairwise
Oregon, Washington, Vancouver Coast and Shelf	marine	—	23	34	11	69
Northern California	marine	—	93	247	43	273
Southern California Bight	marine	—	79	248	28	223
Central California Coast	terrestrial	13,726	161	401	74	905
Central Valley Coast Ranges	terrestrial	24,852	37	75	17	211
Colorado Desert	terrestrial	11,852	18	46	15	165
Great Valley	terrestrial	49,176	67	348	43	567
Klamath Mountains	terrestrial	22,568	37	96	16	261
Modoc Plateau	terrestrial	14,309	19	31	6	40
Mojave Desert	terrestrial	66,832	23	63	12	164
Mono	terrestrial	7,984	15	45	8	129
Northern California Coast	terrestrial	17,135	83	419	51	758
Northern California Coast Ranges	terrestrial	15,524	41	121	22	390
Northern California Interior Coast Ranges	terrestrial	7,494	15	16	10	145
North-western Basin and Range	terrestrial	5,224	7	9	0	0
Sierra Nevada	terrestrial	51,593	67	358	23	511
Sierra Nevada Foothills	terrestrial	18,191	28	87	17	336
Sonoran Desert	terrestrial	12,878	4	6	3	22
South-eastern Great Basin	terrestrial	11,038	7	15	2	42
Southern California Coast	terrestrial	14,473	177	645	77	920
Southern California Mountains and Valleys	terrestrial	27,551	78	340	43	619
Southern Cascades	terrestrial	17,025	28	81	13	212

The first three are marine, followed by the 19 USDA-defined ecoregions.

## Data Records

The CaliPopGen database comprises four datasets, which are hosted at Figshare and can be downloaded as XLSX, TSV and CSV files. For convenience, the life history trait data for both animals (**Dataset 3**^21^) and plants (**Dataset 4**^21^) have also been included in **Dataset 1**^21^ and **Dataset 2**^21^. We combined the genetic and life history data under the assumption that potential users may want to examine correlations between these two classes of data. **Dataset 1**^21^: The Population Genetic Diversity dataset consists of 101 columns, described in Table 1, and is comprised of data from 401 studies on 446 (sub-)species and 4,697 unique species-population-marker type combinations, with the latter equaling the number of rows in the dataset. The first 31 columns summarize taxonomic, population, marker type, and genetic data, while the remaining 70 columns contain data on animal and plant life history (**Dataset 3**^21^ and **Dataset 4**^21^, respectively, see below).

**Dataset 2**^21^: The Pairwise Population Differentiation dataset consists of 106 columns, described in Table 2, and is comprised of data from 199 studies on 197 (sub-)species and 14,703 pairwise population comparisons, with the latter equaling the number of rows. The first 36 columns summarize taxonomic, population, marker type, and pairwise population comparison data, while the remaining 70 columns contain data on animal and plant life history (**Dataset 3**^21^ and **Dataset 4**^21^, respectively, see below).

**Dataset 3**^21^: The Animal Life History Traits dataset consists of 45 columns, containing data for 432 species and subspecies, and is described in Table 3. The first 29 columns describe the life history of species and subspecies, and give details on their conservation status, while columns 30–45 provide information on the sources of these data.

**Dataset 4**^21^: The Plant Life History Traits dataset consists of 37 columns containing data for 177 species and is described in Table 4. The first 23 columns describe the life-history of species and subspecies, and give details on their conservation status, while columns 24–37 provide sources of data. Total species numbers of **Dataset 3**^21^ **& 4**^21^ are higher than the number of species of **Dataset 1**^21^ **& 2**^21^ because we left species in these datasets even though their genetic entries may have been excluded based on the criteria set out in the Methods.

## Technical Validation

### Article classification

During the first step in our screening protocol based on titles and abstracts (see Fig. 4), we examined the repeatability (intra-individual variation), and reproducibility (inter-individual variation) of article classification. Given that multiple individuals were doing the article screening, we recognize that understanding variation at both of these levels is important. During this first screening, six screeners assigned a non-overlapping set of articles into three broad categories (“reject”, “include”, or “possibly include”), based on our five screening criteria (see Methods); we used “possibly include” if it was unclear from the title and abstract if a paper contained appropriate data. Each screener independently evaluated 777–782 articles (total screened = 4,942). To quantify the repeatability of our screeners, all of whom were UCLA undergraduates, each individual re-screened a subset of their original set of articles. 54 randomly selected papers were re-screened by the same person (6 screeners, range 6–13 papers per person, mean = 10.8 papers re-screened/screener). We allowed 10 weeks between the initial and re-screening procedures, which all screeners felt was a sufficiently long time that they would not remember their initial classification, and papers were randomly chosen by the senior authors. To quantify the reproducibility of the screening process across individuals, 421 papers were re-screened by a different individual than the original screener (8 re-screeners, range 46–60 per person, mean = 50.33 re-screened/screener). Each of the 421 papers was re-screened by exactly one new person. This procedure included JB and EMT in addition to the original six undergraduates.

As might be expected, intra-individual repeatability (agreement between the initial and re-screened classification of a paper screened by the same person) was higher than inter-individual reproducibility (agreement between the initial and re-screened classification of a paper screened by two different people): 92.6% (50/54) of papers re-screened by the same individual received an identical score whereas 74.8% (315/421) of papers re-screened by a different individual received an identical score. Across both of these exercises, 17.5% of articles that were re-screened by either the same or different individual (total = 475) were assigned to different categories between the first and second screening. For the inter-individual analyses, 27.7% of “possibly include” articles changed status when screened by different individuals, while only 16.0% of “reject” and 16.3% of “include” decisions changed. However, when we subsequently attempted to extract data during the Data Collection Phase, we did so from both “include” and “possibly include” papers, so the relatively low change of “reject” status makes us comfortable that screener variability and its potential bias had at most a very limited impact in our decision pipeline.

### Data validation

To identify and correct potential recording errors in the datasets after the initial round of data extraction, we flagged numerical outliers and values outside of theoretical expectations for all genetic parameters and life history traits. Both outliers and values outside theoretical bounds may represent values as reported in the original publication, or they may be transcription errors as we compiled datasets. To increase the likelihood of identifying errors via outlier analysis, we examined each genetic parameter distribution separately for each marker type and taxonomic group (for example, *H*_*O*_ of microsatellite markers in Aves was examined separately from *H*_*O*_ of mitochondrial markers in Reptilia), and we examined life history trait distributions separately for each taxonomic group. In all cases, we identified outliers as values greater or less than the upper or lower quartiles +/− 1.5 * IQR (IQR = inter-quartile range), using the function boxplot.stats in the R-package grDevices. For all identified outliers we returned to the original source publication to confirm that values were as reported, or corrected them if they were a data-entry error. Correctly transcribed values falling outside of their theoretical bounds (*H*_*E*_, *H*_*O*_, π, *F*_*ST*_, *G*_*ST*_, *D*_*ST*_ are constrained between zero and 1, *F*_*IS*_ is bounded by −1 and 1, *N*_*e*_ must be greater than zero) were left unaltered, which users of the CaliPopGen databases should consider carefully in using these results.

## Data Availability

The code used to generate figures is available at https://github.com/jbeninde/CaliPopGen. As the data was taken from the published literature manually, no additional code was used to extract data.
